# The effect of high correlated colour temperature office lighting on employee wellbeing and work performance

**DOI:** 10.1186/1740-3391-5-2

**Published:** 2007-01-11

**Authors:** Peter R Mills, Susannah C Tomkins, Luc JM Schlangen

**Affiliations:** 1 Vielife Ltd, 68 Lombard Street, London EC3V 9LJ, UK; 2 Department of Respiratory Medicine, The Whittington Hospital, London N19 5NF, UK; 3 Philips Lighting, Global Organisation Applications Lighting, P.O. Box 80020, 5600JM Eindhoven, The Netherlands

## Abstract

**Background:**

The effects of lighting on the human circadian system are well-established. The recent discovery of 'non-visual' retinal receptors has confirmed an anatomical basis for the non-image forming, biological effects of light and has stimulated interest in the use of light to enhance wellbeing in the corporate setting.

**Methods:**

A prospective controlled intervention study was conducted within a shift-working call centre to investigate the effect of newly developed fluorescent light sources with a high correlated colour temperature (17000 K) upon the wellbeing, functioning and work performance of employees. Five items of the SF-36 questionnaire and a modification of the Columbia Jet Lag scale, were used to evaluate employees on two different floors of the call centre between February and May 2005. Questionnaire completion occurred at baseline and after a three month intervention period, during which time one floor was exposed to new high correlated colour temperature lighting and the other remained exposed to usual office lighting. Two sided t-tests with Bonferroni correction for type I errors were used to compare the characteristics of the two groups at baseline and to evaluate changes in the intervention and control groups over the period of the study.

**Results:**

Individuals in the intervention arm of the study showed a significant improvement in self-reported ability to concentrate at study end as compared to those within the control arm (p < 0.05). The mean individual score on a 5 point Likert scale improved by 36.8% in the intervention group, compared with only 1.7% in the control group. The majority of this improvement occurred within the first 7 weeks of the 14 week study. Substantial within group improvements were observed in the intervention group in the areas of fatigue (26.9%), alertness (28.2%), daytime sleepiness (31%) and work performance (19.4%), as assessed by the modified Columbia Scale, and in the areas of vitality (28.4%) and mental health (13.9%), as assessed by the SF-36 over the study period.

**Conclusion:**

High correlated colour temperature fluorescent lights could provide a useful intervention to improve wellbeing and productivity in the corporate setting, although further work is necessary in quantifying the magnitude of likely benefits.

## Background

Until now the main purpose of indoor lighting has been to aid visually directed tasks in the absence of sufficient external light. There is, however, increasing evidence to suggest that the brightness and wavelength of ambient light is not only important for task completion, but that it can also have strong non-visual biological effects, regulating the human circadian system, and impacting upon the biological clock, mood and alertness.

A number of studies have provided support for the beneficial effects of light, demonstrating a positive influence on vitality, depressive symptoms [1], alertness. [2], psychomotor vigilance and task performance. [3], morning cortisol levels [4], and even sleep quality [5,6]. Additionally, bright-light exposure during winter appears to be effective at improving health-related quality of life and alleviating distress [7]. Exposure to bright light in the morning and evening in the workplace has also been shown to improve self-reported mood, energy, alertness and productivity in individuals with "sub-syndromal seasonal affective disorder" [8].

The recent discovery of 'non-visual' retinal receptors has confirmed an anatomical basis for the observed biological effects of light, with the photopigment melanopsin playing an essential role in phototransduction [9]. As such, light has a broad regulatory impact on human physiology within virtually all tissues in the body with action spectra in humans showing the peak sensitivity for these effects to be in the short wavelength portion of the spectrum. [10,11].

It has been suggested that the relative shortage of daylight exposure for office workers during daily life may compromise their health and wellbeing, which in turn has stimulated interest in the applications of light in the corporate setting. Of particular relevance is the fact that whilst outdoor illuminance typically ranges between 2000 and 100,000 lux, indoor office illuminance is usually considerably lower, with norms of approximately 500 lux. Moreover, typical fluorescent indoor lighting contains considerably less short wavelength "blue spectrum" light than natural daylight, precisely the component of the spectrum thought to be highly relevant for achieving non-visual, biological effects.

The amount of blue light in the spectrum of light sources increases with increasing colour temperature. So far a number of studies have investigated the effects of the colour temperature of lighting on mental activity, the central nervous system and alertness. These studies have demonstrated that higher colour temperatures (7500 K versus 3000 K) are more activating from the viewpoint of mental activity level [12]. Both the parasympathetic and sympathetic nervous systems are thought to be enhanced under higher colour temperature conditions. [13] and drowsiness has been observed to be higher under lower colour temperature lighting when comparing 3000 K with 5000 K [14].

Whilst findings of previous studies have been encouraging, these have been based on very small sample sizes and generally conducted within carefully controlled laboratory type environments. There is currently little understanding of the effect of lighting conditions outside such a setting, such as in the workplace. The current study addresses this issue, at least in part, with its relatively large sample size and the fact that it was conducted in a 'real world' workplace setting.

Understanding of the action spectra of many non-visual, biological effects remains far from comprehensive. Nocturnal melatonin suppression is probably the most frequently studied non-visual, biological effect of light. Its action spectrum is well established and appears to be most sensitive to short wavelength light. [10,11]. Also, in achieving phase advancing [15] or alerting effects. [16,17], short wavelength light is reported to be more effective as compared to longer wavelength light. It is therefore reasonable to assume that a first estimate of the non-visual effects of a light source can be derived from the action spectrum for nocturnal melatonin suppression. Using this assumption,17000 K lamps would be expected to be 1.55 times as effective as compared to daylight at equal illuminance in achieving non-visual biological effects, and in comparison to standard low colour temperature lighting of 3000 K could be expected to be 2.4 times as effective. With this background information in mind, it can be postulated that the new high correlated colour temperature lights would have significant effects upon feelings of wellbeing, alertness, concentration and possibly work performance in those exposed to it.

The aim of this study was to quantify the effects of newly developed high correlated colour temperature fluorescent lighting on functioning, well-being and work performance of individuals working within a call-centre.

## Methods

A prospective, controlled intervention was conducted involving study participants working as call-handlers on two floors of the offices of Standard Life Healthcare (SLH) in Stockport, UK. SLH is a shift-working call centre with long working days spanning 8 am–8 pm, divided into early and late shifts. The two floors used for the study are identical in their layout and operational function within the organisation. Each floor is equipped with 600 mm square recessed luminaries with aluminium louver (4 × 6 cells) and four 18 W fluorescent tubes. The luminaire spacing is 2.4 m × 2.4 m. Each work area has dark floors and white walls. Floors have windows on approximately 80% of both their East and West wall areas. Blinds are present and used in such a way that typically more than 50% of the window area is covered.

**Figure 1:**
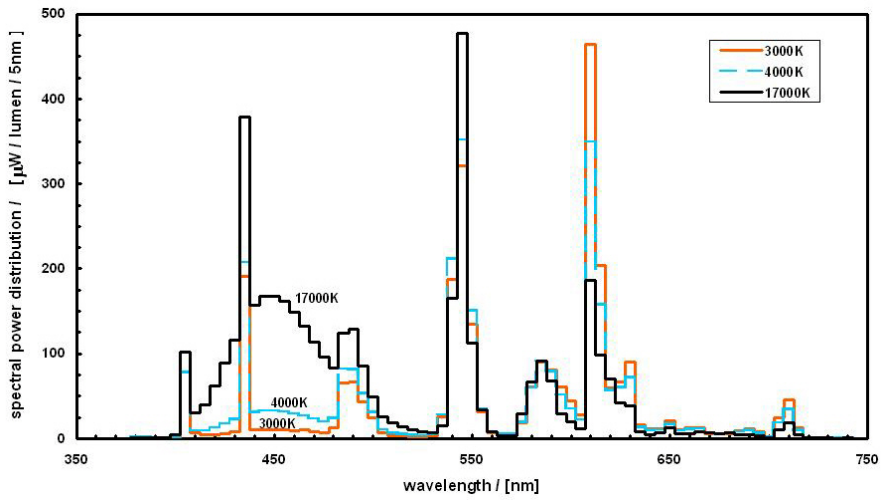
**Spectral power distribution of the high (17000 K) correlated colour temperature lamps**. The spectral power distributions of typical 3000 K and 4000 K fluorescent lamps are plotted for comparison.

At baseline both floors were illuminated with lights with a correlated colour temperature of 2900 K. Throughout the study, the lighting on one floor (floor B) was unchanged, with employees working on this floor being used as the control group. On the other floor (floor A), a lighting intervention was implemented after baseline measurements. The intervention involved a lamp change to the entire lighting system on this floor, with all existing fluorescent lamps being replaced by new high correlated colour temperature fluorescent lamps (ActiViva Active, Philips). These lamps contain an enhanced amount of short wavelength light with a resulting higher colour temperature of 17000 K. Figure 1 shows the spectral power distribution of the new lamps. The lamp change occurred on a non working day (Sunday) and participants were not informed of whether theirs was the intervention or control arm of the study.

Before the lamp change, horizontal and vertical illluminance values were determined on more than 10 desks from each floor, deemed representative of the range of working conditions. The horizontal illuminance was measured at the working plane (desk surface). The vertical illuminance was measured at the eye position, when sitting behind the desk. After the lamp change the illuminance values at each desk were measured once with artificial lighting switched on, and once with it switched off. This allowed estimation of the daylight contribution to the indoor illuminance. All measurements were taken at noon on a cloudy winter day.

Participation in the study was voluntary; those who did not wish to participate were offered seating in a different part of the building. Individuals were informed that the aim of the study was to assess the acceptability of a new type of indoor lighting. They were informed that the lighting would be changed on both floors and that they would be required to complete online questionnaires during the 14 weeks of the study. Participants were not informed that the aim of the study was to assess any particular aspect of work performance or wellbeing and were not told which floor would receive the new lighting technology. All participants digitally accepted the terms and conditions and provided their consent to participate in the study prior to completing the questionnaires.

The lights used in the study have passed all appropriate European Union safety standards and are in general production. We were informed by the Central Office for Research Ethics Committees in the UK that ethical committee approval was not required for this study.

### Data collection

Individuals' alertness, performance, concentration and health related quality of life were assessed by means of two online questionnaires. The questionnaires were completed on three separate occasions, at baseline (week 1, 7–11^th ^February 2005), week 7 and week 14. Questionnaires completion took approximately 20 minutes and was done at one sitting during the working day.

The first questionnaire was a modification of the Columbia Jet Lag Scale [18], originally designed to quantify changes in alertness, memory, fatigue and general wellbeing associated with crossing time zones. Although the current study was not assessing jet lag, the majority of the constructs quantified by the questionnaire was relevant to the shift-working practices of the call centre. Nine of the eleven items of the questionnaire were derived directly from the original instrument with possible answers of (i) not at all, (ii) a little bit, (iii) moderately, (iv) quite a bit and (v) extremely. Scores were attributed to each item from 1 to 5 depending upon the response (1 = not at all to 5 = extremely) for a combined overall score out of 45. A high score indicated significant issues with alertness, lethargy, sleepiness and concentration and a low score indicated few or no issues. The nine items were:

Over the last 3 days how much have you been bothered by:

1. Fatigue or tiring easily

2. Trouble concentrating or thinking clearly

3. Physical clumsiness

4. Decreased daytime alertness

5. Trouble with memory

6. General feelings of weakness

7. Light headed, dizzy, or other uncomfortable sensations in the head

8. Lethargy and sluggish feelings

9. Sleepiness during the day

The other two items were (i) self assessed job performance, which was derived from the World Health Organisation Health and Work Performance Questionnaire (WHO-HPQ) and (ii) self assessed overall alertness and concentration:

10. On a scale of 1 to 10, where 1 is the worst performance anyone could have at your job and 10 is the performance of a top worker, how would you rate your overall performance over the last 3 days?

11. On a scale of 1 to 10, where 1 is not alert at all and 10 is fully alert. All things considered, how alert and able to concentrate have you been over the last 3 days?

The second questionnaire was the short form 36 (SF-36) health related quality of life instrument with standard scoring performed according to the published literature [19]. Only certain items from this questionnaire were of particular interest to this study; however, the questionnaire was administered in its entirety in order to avoid introducing bias in responses given to this previously well validated instrument [20]. On final questionnaire completion, at the end of the study, participants on both floors were asked to comment about the lighting on their floor. Specifically, they were asked whether it was liked or disliked and whether they wished to keep the current lighting or revert to previous lighting conditions.

### Data analysis

Digitally collected data were transferred to STATA version 8.2 for analysis. All datasets were checked for outliers and errors to ensure that all responses fell within the expected range of values prescribed by the two questionnaires. Coding of SF-36 items and derived measures was conducted according to validated literature guidelines. [21]. A combined measure was derived by summing the initial 9 items in the modified Columbia jet lag scale yielding a maximum possible score of 45 and a minimum of 9. All data collection and storage was compliant with the UK Data Protection Act 1998.

All items from the modified Columbia Jet Lag Scale were utilised in the analysis as these were all measures relevant to the principle aims of the study, i.e. workplace functioning, wellbeing and work performance. Five of the SF-36 combined measures were utilised in the analysis (General Health, Vitality, Social Functioning, Role Emotional and Mental Health), as again these were the constructs considered relevant to the main study aims.

The distribution of variables by floor and the mean score for each item was obtained for the range of measures described above. The selected SF-36 combined measures were compared with US norms. [21] in order to assess the generalisability of findings.

Two-sided unpaired t-tests were used to compare baseline characteristics between the control and intervention floors. Significance was obtained on 67 degrees of freedom (d.f.). The within-floor improvements over the study period were ascertained by examination of percentage mean improvement by group compared to baseline scores, and by using two-sided paired t-tests on 22 and 45 d.f. respectively. Finally, two-sided unpaired t-tests were used to examine whether there was a statistically significant difference in individual scores at the end of the intervention period in the two groups, controlling for individual baseline scores. A total of thirty questionnaire items or scores were examined from the two questionnaires, and a Bonferroni correction for Type I errors was accordingly applied to each set of tests based on this total number when interpreting the significance of the t-statistics obtained: the threshold t-statistic used to determine significance was deemed equal to probability divided by 30, the total number of questionnaire items examined.

## Results

Sixty-nine individuals agreed to take part in the study (23 on the control floor and 46 on the intervention floor), representing 49% of the total eligible population during the study period. There were no significant differences in the distribution of participants by gender or age by floor (X^2 ^= 0.30, p = 0.58; X^2 ^= 0.04, p = 0.84 respectively).

No statistically significant differences were observed between horizontal or vertical desk illuminance between the two floors at baseline. Differences between mean horizontal and vertical illuminance between the two floors after the lamp change were similarly non-significant (Table 1). The estimated daylight contribution to the illuminance is also documented in Table 1. The average daylight contribution to the horizontal illuminance is small (average 12%), but studied daylight contributed between 40% and 55% to the vertical illuminance.

**Table 1:** Horizontal and vertical desk illuminance values on the intervention and control floors.

Floor	Average horizontal illuminance at working plane (SD)	Average % daylight contribution to horizontal illuminance (SD)	Average vertical illuminance at the eye position (SD)	Average % daylight contribution to vertical illuminance (SD)
A. Intervention (17000 K)	311 lux (112)	13% (10)	170 lux (85)	40% (33)
B. Control (2900 K)	354 lux (45)	11% (10)	128 lux (44)	55% (32)

Analysis of the distribution of variables and between group t-tests at baseline showed no significant difference in composite scores or responses to individual items between the two floors. Tables 2 and 3 show the mean scores derived from the modified Columbia Scale and the SF-36 questionnaire at baseline and study end for both the intervention and control groups. In addition the results of between and within group t-tests are also documented.

**Table 2:** Distribution of Modified Columbia Scale scores examined by group and time period.

		Baseline		Study End (3 months)		% change		Unpaired t test (df = 67)	Paired t test (df = 22)	Paired t test (df = 45)	Unpaired t test (df = 67)
Item No.	Description	Control (n = 23)	Intervention (n = 46)	Control (n = 23)	Intervention (n = 46)	Control	Intervention	Baseline comparison	Control change	Intervention change	3 month comparison
			
		mean (SD)	mean (SD)	mean (SD)	mean (SD)			t statistic	t statistic	t statistic	t statistic

1	Fatigue/tiring easily?	2.9 (0.9)	2.9 (1.1)	2.7 (1.1)	2.1 (1.0)	7.6	26.9	-0.16	0.93	4.04**	1.76
2	Trouble concentrating	2.5 (1.1)	2.9 (1.2)	2.5 (0.9)	1.8 (0.9)	1.7	36.8	-1.23	0.21	5.84**	3.46*
3	Physical clumsiness?	2.0 (1.1)	1.7 (1.0)	1.6 (0.6)	1.5 (0.9)	21.7	13.7	1.02	1.93	1.48	-0.70
4	Decreased daytime alertness?	2.7 (1.1)	2.5 (1.2)	2.1 (0.8)	1.8 (0.8)	21.0	28.1	0.74	2.73	3.96**	0.45
5	Trouble with memory?	2.4 (1.3)	1.9 (1.1)	2.1 (0.8)	1.5 (0.7)	12.5	21.3	1.65	1.07	2.80	0.38
6	General feelings of weakness	2.0 (1.1)	2.0 (1.0)	1.7 (0.7)	1.4 (0.8)	17.0	26.7	0.33	1.56	3.60*	0.67
7	Light-headed & dizzy	2.2 (1.4)	2.1 (1.3)	2.0 (1.1)	1.4 (0.8)	9.8	33.7	0.26	1.10	4.04**	1.74
8	Lethargy/sluggish feelings?	3.0 (1.0)	2.7 (1.3)	2.3 (0.8)	1.8 (0.8)	23.5	31.7	0.93	3.43*	5.07**	0.55
9	Sleepiness in day	3.0 (1.2)	2.8 (1.2)	2.6 (1.0)	1.9 (0.7)	14.5	31.0	0.66	2.47	4.90**	1.55
10	Work performance	7.0 (1.7)	6.4 (1.5)	7.3 (1.6)	7.6 (1.4)	4.4	19.4	1.45	-1.16	-6.07**	-2.72
11	Alertness and concentration	6.2 (1.8)	6.1 (1.9)	6.8 (1.7)	7.5 (1.8)	9.9	22.9	0.23	-2.13	-4.34**	-1.57
	Combined Score (first 9 items)	22.7 (7.5)	21.5 (8.3)	19.5 (5.2)	15.4 (5.7)	14.3	28.6	0.59	2.60	5.22**	1.53

* = p < 0.05, ** = p < 0.001 equivalent after applying Bonferroni correction

**Table 3:** Distribution of selected SF-36 combined measures by group and time period.

		Baseline		Study End (3 months)		% change		Unpaired ttest (df = 67)	Paired ttest (df = 22)	Paired ttest (df = 45)	Unpaired ttest (df = 67)
Item No.	Description	Control (n = 23)	Intervention (n = 46)	Control (n = 23)	Intervention (n = 46)	Control	Intervention	Baseline comparison	Control change	Intervention change	3 month comparison
			
		mean (SD)	mean (SD)	mean (SD)	mean (SD)			t statistic	t statistic	t statistic	t statistic

GH	General Health	65.5 (22.4)	67.8 (20.5)	70.3 (22.0)	73.8 (17.0)	7.4	8.7	-0.44	-1.57	-2.33	-0.25
V	Vitality	43.2 (23.5)	48.4 (20.4)	50.5 (19.0)	62.1 (17.1)	17.0	28.4	-0.94	-1.95	-4.44**	-1.25
SF	Social Functioning	63.6 (27.7)	75.0 (25.4)	81.0 (19.5)	85.6 (17.7)	27.4	14.1	-1.71	-3.35*	-3.09	1.11
RE	Role Emotional	77.5 (29.1)	80.8 (23.8)	80.4 (27.9)	86.1 (19.5)	3.7	6.5	-0.50	-0.56	-1.53	-0.39
MH	Mental Health	62.8 (22.2)	64.3 (20.5)	67.8 (16.8)	73.3 (15.8)	8.0	13.9	-0.28	-1.78	-3.42*	-0.93

* = p < 0.05, ** = p < 0.001 equivalent after applying Bonferroni correction

The mean baseline SF-36 derived scales were compared to published reference scores from a normal US population sample. [21]. Of the five SF-36 scales utilised in the study, the mean scores obtained from our study population were significantly different in three instances: the study population reported worse health status in (i) vitality, (ii) social functioning and (iii) mental health compared to the reference US population (respective two-sided t-tests and p-values after application of Bonferroni correction: -5.60 and p < 0.001; -4.34 and p < 0.001; -4.92 and p < 0.001). The remaining selected SF-36 scales (role emotional and general health) were not significantly different.

Following the three month intervention period, exploration of within-group improvements in the intervention group showed substantial and significant improvements in a number of areas. In contrast, significant differences over time were found for a smaller range of variables within the control group, with the magnitude of observed differences tending to be less. Of interest were those variables for which a statistically detectable improvement was observed in the intervention group, but not in the control group. In general those individuals exposed to the new lighting technology showed a consistent improvement in the areas of fatigue, concentration, memory, mood and energy as compared with individuals who did not have a lighting change (see Table 4). Improvements of 30% or more compared to baseline measures were observed in the areas of (i) concentration, (ii) light headedness, (iii) lethargy and (iv) sleepiness in the intervention group. In addition, the intervention population showed significant improvements in two of the five investigated SF-36 scales at study end (vitality and mental health) compared to baseline scores, which for vitality was highly significant (p < 0.001). In contrast, the control group only showed borderline significant improvement on the social functioning scale (Table 4).

**Table 4:** Areas of substantial improvement in the intervention group compared to baseline measures (where a concomitant improvement was not observed in the control group).

Area	Description	Percentage Improvement over baseline measure
Fatigue	Item of original Columbia Scale	26.9%
Concentration	Item of original Columbia Scale	36.8%
Daytime Alertness	Item of original Columbia Scale	28.1%
Feelings of Weakness	Item of original Columbia Scale	26.7%
Light-headedness	Item of original Columbia Scale	33.7%
Sleepiness	Item of original Columbia Scale	31.0%
Work Performance	Additional item (derived from WHO-HPQ)	19.4%
Alertness & oncentration	Additional item	22.9%
Vitality	Combined measure from SF-36	28.4%
Mental Health	Combined measure from SF-36	13.9%

**Figure 2:**
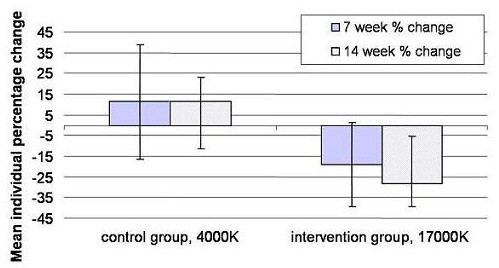
**Percentage change in reported 'trouble concentrating or thinking clearly' during the preceding 3 days**. The plot shows data from 7 and 14 weeks after baseline in control and intervention groups (error bars represent 95% confidence interval for the mean change).

Between group comparisons at study end, controlling for baseline differences, showed the intervention group had significantly better status in the area of concentration (item 2 of the Columbia Scale) (p < 0.01 after Bonferroni correction for Type I errors). The chronology of the observed improvements in concentration was investigated further by additionally analysing the seven week data for this item at an individual level, in order to ascertain how rapidly the observed improvements had occurred. Table 5 shows the mean data by group at each time period with Figure 2 showing the mean individual percentage change for this item at week 7 and week 14 as compared to baseline. The observed change in the control group was in a negative direction (shown on the graph as an increase in score), with little difference between scores at week 7 or week 14. Conversely, the reduction in scores in the intervention group reflected an improvement in this measure, much of which had already occurred by week 7. The changes in the two groups is reflected by two-sided unpaired t-tests that explore the difference between the groups at each time period and are significant both at week 7 and week 14. (t = 2.48, p = 0.02 at week 7; t = 3.46, p = 0.001 at week 14).

**Table 5:** Trouble concentrating or thinking clearly at baseline, week 7 and week 14 by group. Mean score derived from the Columbia Scale according to response to the second question, "Over the last 3 days how much have you been bothered by trouble concentrating or thinking clearly? Possible answers and scoring: 1 = not at all, 2 = a little bit, 3 = moderately, 4 = quite a bit, 5 = extremely.

Time point	Control (n = 23)	Intervention (n = 46)
	mean score (SD)	mean score (SD)

Baseline	2.5 (1.1)	2.9 (1.2)
Week 7	2.6 (1.1)	1.9 (0.9)
Study end (Week 14)	2.5 (0.9)	1.8 (0.9)

Although not reaching statistical significance after Bonferroni correction, between group analysis of self-reported work performance over the 3 months of the study showed a sizeable positive trend in favour of the intervention. Within group analysis yielded an almost 20% increase in mean work performance score in the intervention group, with only marginal changes seen within the control group, suggesting this area would warrant further exploration in future studies.

## Discussion

The present study is the first to investigate the ability of newly developed 17000 K fluorescent lights to achieve non-visual, biological effects within a workplace setting. Despite having had a relatively large number of participants compared to existing research in the field, the total number taking part in the study was still small. This, together with the fact that there was an uneven distribution of subjects in the two experimental groups makes drawing firm conclusions difficult. If the control arm of the study contained a greater number of individuals it is possible that the within group analysis for this group would have yielded more significant results, akin to those seen in the intervention arm. Certainly the direction of observed changes within the control arm was often in the same direction as that seen within the intervention arm.

A further consideration is that this was not a fully blinded study. Although participants were not explicitly told to which group they were assigned, feedback indicated that the lighting differed visually between the two floors. There is hence the possibility of biased responses to questionnaires by those on either floor and cross contamination of information between the groups. There is also a possibility of bias in responses due to the Hawthorne effect [22,23] and indeed, the placebo response is well recognized within lighting studies. [24,25]. Although every effort was taken to ensure study participants were not influenced as to the possible outcomes of the study, this cannot be fully discounted. As it is not possible to quantify the extent of such bias, any difference in the relative improvements of indicators between groups must be interpreted with care. This having been said, this was a 'real world' study designed to ascertain whether the positive effects of high correlated colour temperature lighting observed in a more controlled environment could be translated to the workplace.

Of note, our sample differed at baseline in a number of SF-36 measures from a general US population. [21], with a tendency for lower scores on some of the scales indicating poorer self-reported health. This modest difference is not unexpected, given the shift working nature of study participants, and serves to highlight the health and wellbeing issues experienced by shift-workers in call centres.

Since the study was conducted from February to May, some of the improvements observed may be attributable to seasonal effects, associated with the lengthening of days during the transition from winter to spring. There were indeed improvements observed in both groups for a number of measures, and improvements in the control group may provide an indication of the magnitude of the seasonal effects on the measures collected here. However, it is reassuring that there was a statistically significant difference between groups for a key measure at study end, which can reasonably be attributed to the effects of the intervention, beyond any seasonal effects. It is encouraging that findings indicate improvements in a number of self-reported measures including aspects relating to concentration, alertness and energy.

It appears that the lights contributed to general feelings of well-being, which may plausibly have led to the observed self-reported improvements in work performance. For the duration of the study SLH provided weekly group call handling data for both floors, and although this was not individual-specific, and hence not amenable to robust statistical analysis, it did show a modest improvement in the proportion of incoming calls answered from week 9 until the end of the study in the intervention group as compared to the control group (0.53%) which within the context of a large call-handling centre could lead to significant improvements in customer satisfaction.

The present study did not investigate the effect of the light intervention on the sleep quality of participants directly; however, it is possible that some of the observed effects were associated with an improvement in sleep quality. Exposure to bright light during the daytime has been reported to enhance nocturnal melatonin levels [26,27] and improve sleep [28,29] and although the present study did not use bright light conditions, the larger amount of short wavelength light in the high correlated colour temperature light sources used for the intervention may have resulted in an ambience more analogous to the lighting conditions outdoors. It is certainly feasible that compared to conventional light sources, lamps with enhanced short wavelength composition may be used to reduce the light levels needed for achieving biological, non-visual effects so that these effects can be realised in an energy efficient way. With greater awareness of environmental issues and energy consumption globally, this is an area that should be investigated further.

Feedback from study participants indicated that the new lighting was well tolerated, compared with the standard lighting, and was preferred by the majority of individuals. Most pertinently, feedback indicated that the majority of participants on the intervention floor (41 of the 46) wished to keep the new lights at the end of the intervention period. The specific wellbeing effects of the new lighting found in this study probably explain, at least in part, the high acceptance of these lights.

The questionnaires used in this study are not specifically designed to evaluate the effect of lighting interventions in the workplace; however, certain individual questions reported in this paper appear informative in this context, and on this basis we would recommend questions from the Columbia Jet Lag scale, and a selection of the scales from the SF-36 questionnaire, for future evaluation of lighting conditions.

Knowledge about potential health and well-being related benefits of light has led to an understanding of the need for indoor lighting strategies that are optimal for vision and human physiology simultaneously. Exposure to the new generation 17000 K industrial lights in a call centre in Stockport resulted in positive trends observed across a wide range of wellbeing and functional status variables, as compared to a control population, as well as a significant improvement in reported ability to concentrate.

## Conclusion

The installation of new high correlated colour temperature (17000 K) fluorescent lighting in a shift-working call centre appears to have contributed to wide ranging improvements in wellbeing, functioning and work performance amongst study participants. The lighting is well tolerated and has the potential to be a cost-effective means of impacting upon employee wellbeing and productivity. Further studies are needed to quantify the observed effects in larger and different working populations.

## Abbreviations

SF-36: Short Form 36 Questionnaire

WHO-HPQ: World Health Organisation Health and Work Performance Questionnaire

SLH: Standard Life Healthcare

## Competing interests

LS is an employee of Philips, which provided the lights for the study.

## Authors' contributions

PM developed the study protocol, collected the data and contributed to data analysis and writing of the manuscript.

ST analysed the data and contributed to writing the manuscript.

LS organised the follow-up of the lighting intervention and contributed to data analysis and writing of the manuscript.
